# ATTR Epidemiology, Genetics, and Prognostic Factors

**DOI:** 10.14797/mdcvj.1066

**Published:** 2022-03-14

**Authors:** Chukwuemeka A. Obi, William C. Mostertz, Jan M. Griffin, Daniel P. Judge

**Affiliations:** 1 Division of Cardiology, Medical University of South Carolina, Charleston, South Carolina, US; 2 Division of Cardiology, Columbia University Irving Medical Center, New York, New York, US

**Keywords:** transthyretin, misfolding, ATTR amyloidosis, mutation, prognosis

## Abstract

Transthyretin amyloid cardiomyopathy (ATTR-CM) is an underdiagnosed disease and an underestimated cause of both heart failure and conduction abnormalities. It is characterized by pathologic accumulation of extracellular protein arising from unstable transthyretin (TTR) tetramers, which dissociate into monomers that misfold, aggregate, and form insoluble fibrils that are resistant to proteolysis. Cardiac amyloidosis appears in two distinct forms: hereditary and wild-type. There is considerable heterogeneity in the clinical presentation of ATTR, ranging from primarily cardiac, primarily neuropathic, or mixed cardiac and neuropathic disease. Pathogenic variants in the *TTR* gene that predominantly involve the heart include Val122Ile, Leu111Met, and Ile68Leu. The wild-type form of ATTR is also predominantly cardiac. Phenotypic heterogeneity is linked to differences among specific pathogenic *TTR* variants, geography, and the subtype of endemic versus nonendemic disease. Factors contributing to wild-type ATTR are largely unknown, but similar factors likely influence the penetrance of hereditary ATTR. Recognition of ATTR-CM is improving due to the increased use of cardiac scintigraphy as a noninvasive diagnostic tool, and early recognition of cardiac infiltration is crucial to optimize long-term prognosis.

## Introduction

Transthyretin amyloid cardiomyopathy (ATTR-CM) is characterized by pathologic accumulation of unstable extracellular tertiary structures that misfold, aggregate, and form insoluble fibrils in blood vessels, bones, and major organs.^1,2^ Transthyretin (TTR) misfolding and subsequent deposition in tissues are facilitated by TTR mutations that decrease the stability of the normally folded protein.^3^

ATTR occurring in heart muscle (ATTR-CM) is an underdiagnosed disease and underestimated cause of heart failure (HF), conduction abnormalities, and cardiac arrhythmias.^4^ TTR misfolding can lead to two distinct forms of cardiac amyloidosis: hereditary (ATTRv) and wild-type (ATTRwt).^5^ In ATTRv, an amyloidogenic mutation in the *TTR* gene facilitates the dissociation of its tetramer into monomers and promotes subsequent misfolding.^6^ Amyloid formation also occurs in the absence of pathogenic variants in *TTR*, when dissociated TTR monomers misfold and aggregate into amyloid fibrils in the presence of favorable conditions, such as aging and oxidative stress in ATTRwt.^7,8^ Misfolded TTR oligomers in circulation cause progressive interstitial infiltration, leading to increased oxidative stress and mitochondrial damage, increased cardiac wall thickness, and diastolic dysfunction.^9,10,11^ This may accelerate the progression of ATTR-CM since worsening HF amplifies conditions that may lead to additional TTR misfolding and deposition.

## Epidemiology and Distribution of ATTR Amyloidosis

The true incidence and prevalence of ATTR-CM are currently unknown.^12,13^ Historically, ATTR-CM has been considered a rare disease that mainly affects the elderly.^14^ This perception is partly due to the phenotypic heterogeneity of the disease, ranging from exclusively neurologic involvement to cardiac-restricted disease.^15^ This variability results from differential effects of various *TTR* mutations, their occurrence in an endemic versus nonendemic location, genetic and demographic factors such as age and sex, and probably other environmental factors that are currently unknown.^15^ However, recognition of ATTR epidemiology is evolving due to the increased use of cardiac scintigraphy as a noninvasive diagnostic tool.^1^ For example, using cardiac scintigraphy, Castaño et al. discovered that roughly 13% of patients admitted for HF with preserved ejection fraction (HFpEF) and a wall thickness > 12 mm may have ATTRwt, and approximately 16% of patients with severe calcific aortic stenosis (AS) undergoing percutaneous transcatheter aortic valve implantation have ATTR-CM.^16^ Furthermore, carpal tunnel syndrome, which affects 3% to 6% of the general population, may precede a diagnosis of ATTR-CM by approximately 6 years.^17^ This raises the question of whether ATTR-CM should be considered a common condition despite poor recognition.^13^

The US Congress, through the Rare Disease Act of 2002, defines rare disease as fewer than 200,000 affected individuals in the United States, whereas the Public Health section of the European Commission defines rare disease as affecting fewer than 1 in 2,000 people in Europe. The prevalence of ATTR-CM may be substantially higher than these numbers,^18^ especially given that the prevalence of HF in the US is around 6.2 million per the Centers for Disease Control and Prevention.^19^ Approximately 50% of people with HF have a preserved EF (HFpEF), and transthyretin deposition is found on autopsy in about 25% of those over 80 years of age with HFpEF. In a cohort of 109 individuals with an antemortem diagnosis of HFpEF without known amyloid who underwent autopsy, 5% had robust ATTR deposition in their hearts, while 12% had more mild cardiac deposition.^20^ This suggests that at least 155,000 people (5% of estimated HFpEF prevalence) have ATTR, but milder deposition is probably playing a role in many others with HFpEF. Additionally, several studies indicate disparity in the racial prevalence of HF,^21^ suggesting that the prevalence of ATTR among non-Whites with HFpEF may be higher than among Whites.

## Genetics

Transthyretin is a tetrameric serum transport protein encoded by the *TTR* gene on chromosome 18q12.1, and it spans 4 exons.^22^ Traditional numbering of *TTR* codons came from early amino acid sequence analysis of the circulating protein, which lacks an N-terminal signal peptide of 20 residues.^23^ Later analysis of the DNA sequence helped to determine the full-length proprotein.^24^ Based on proper nomenclature by the Human Gene Organization (HUGO), codon numbering should begin with methionine—which adds 20 residues to the traditional numbering that arose from early serum protein analysis—as well as three-letter abbreviations for amino acids. This manuscript follows the majority of published literature on ATTR, using the traditional numbering and three-letter abbreviations for amino acids. However, clinical genetic laboratories typically use HUGO-based nomenclature. For example, a common pathogenic variant in *TTR* may be designated as V30M or Val30Met by traditional annotation, or p.Val50Met by HUGO-based nomenclature.

**Figure 1:**
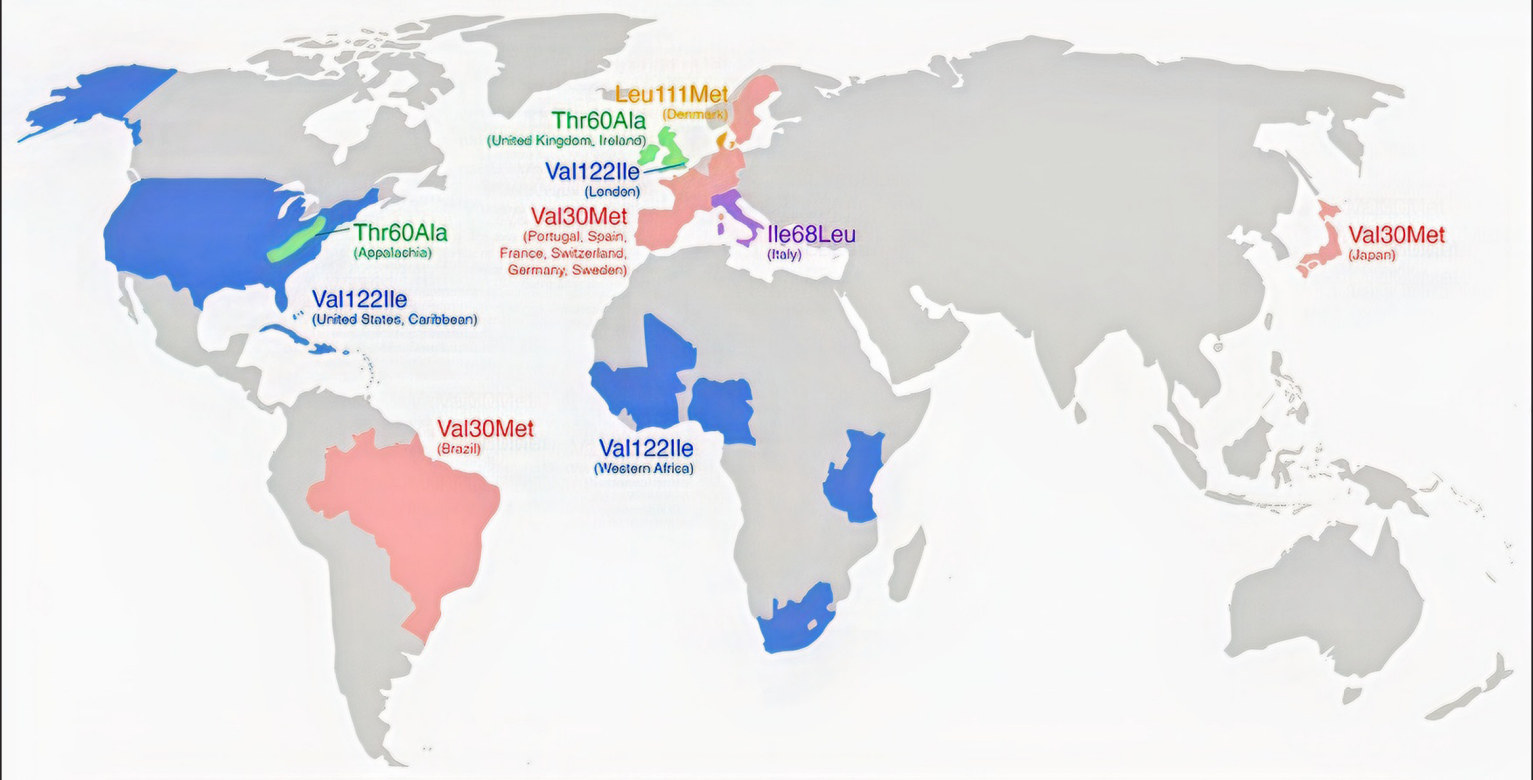
Worldwide distribution of most common ATTRv genotypes by country/region. Countries and regions with relatively high levels of *TTR* pathogenic variants noted in the text are represented by different colors. Each *TTR* variant is designated by its traditional nomenclature. Most *TTR* variants have even more regional heterogeneity in these countries and regions. ATTRv: hereditary transthyretin amyloid; TTR: transthyretin

More than 130 pathogenic variants in *TTR* related to familial ATTR have been reported.^25^ Heterozygosity is enough to prompt disease manifestations, although reduced penetrance and variable expression make family history an unreliable factor in recognizing ATTRv except among individuals and families with early-onset ATTRh.^26^ These mutations tend to cluster into geographic or ethnic groups with an autosomal-dominant pattern of inheritance.^10^ In the US, the most common *TTR* mutations are Val122Ile, Thr60Ala, and Val30Met, whereas the most common *TTR* mutations worldwide are Val30Met, Val122Ile, and Glu89Gln.^6,27^ Val30Met may be the most commonly recognized mutation worldwide, mostly found in regions of Portugal, Spain, France, Japan, Sweden, South America, and some nonendemic parts of Africa.^6^ On the other hand, Val122Ile originated in West Africa and is found in approximately 3% to 4% of African Americans, with cardiac manifestations occurring in later stages of life.^28^ Although the worldwide prevalence of the Val122Ile variant may exceed that of the Val30Met variant, the penetrance of Val30Met appears higher than for Val122Ile, and the incidence of ATTRv related to Val30Met may be highest.^6,29^ Leu111Met and Ile68Leu are mutations mostly reported in Denmark and Italy, respectively, and cause severe cardiomyopathy in relatively early ages.^30^ The Thr60Ala variant is the second most-common TTR variant in the US and the most common in the UK and Ireland, affecting approximately 1% of the population of north-western Ireland.^31,32^ Val122Ile is known to be the most common cause of late-onset ATTR-CM in the US.^33^ Other common causes of ATTRv-CM include Thr60Ala (early-onset phenotype), Ile68Leu (late-onset phenotype) in Italy, and Leu111Met (early-onset phenotype) in Denmark.^13,34^ Worldwide distribution of most common ATTRv genotypes by country/region is shown in ***Figure 1***.

There is heterogeneity in disease phenotypes, ranging from predominantly neuropathic (familial amyloid polyneuropathy, or TTR-FAP), predominantly cardiac (TTR amyloidosis cardiomyopathy, or ATTR-CM), or a mixed phenotype.^4^ In addition, an oculoleptomeningeal type with symptoms predominantly involving the central nervous system has been reported with some rare mutations.^35^ According to an international registry, Transthyretin Amyloidosis Outcomes Survey (THAOS), most patients enrolled from the US are elderly men with wtATTR-CM and a cardiac-predominant phenotype compared with patients enrolled in this registry from Europe.^27^ ATTRwt tends to produce a predominantly cardiac phenotype. The predominant phenotype in patients with hereditary ATTR varies based on the underlying mutation. Both Val122Ile and Leu111Met typically cause cardiomyopathy. However, there also can be a substantial component of neuropathy in patients with Val122Ile, with almost 60% of these individuals in the THAOS survey reporting sensory neuropathy.^27^ In another study with a mixed population of self-reported White and Black individuals with Val122Ile, 20% to 27% reported neuropathy.^36^ Early-onset Val30Met predominantly causes polyneuropathy, but late onset often has cardiac involvement.^6^ Glu89Gln has a mixed phenotype of polyneuropathy and cardiomyopathy.^6,36^

Heterogeneity in disease penetrance also occurs across different geographic regions. For example, age of disease onset related to Val30Met is much later in Sweden than in Portugal and Japan, and the Portuguese population has 80% higher penetrance of the Val30Met mutation during mid-age (50 years) compared with the French population.^37^ In one study evaluating a potential parent-of-origin effect, the authors compared age of onset of TTR-FAP among French and Portuguese families with the Val30Met pathogenic variant. Out of 33 Portuguese families, the transmitting parent was found to be the mother in 153 individuals and the father in 138.^38^ In comparison, out of 48 French families, the transmitting parent was the mother in 216 individuals and the father in 219. In the Portuguese families, phenotypic manifestations were earlier when the pathogenic TTR variant was inherited from the mother instead of the father (*P* < .002). The age of onset of TTR-FAP was not significantly different in the French families when comparing maternal and paternal transmission of the *TTR* mutation.

Variability among patients in terms of age of disease onset also may depend on the phenotype.^39^ Traditionally, TTR-FAP and ATTRv-CM were considered two distinct disorders, although ATTR is now considered to be a single disease with a spectrum of manifestations, including nearly exclusively neuropathy or cardiomyopathy, or (more commonly) a combination of these and other manifestations. In prototypical TTR-FAP, signs and symptoms develop most commonly in the third to fifth decade of life but can be noticed from the second decade of life. Penetrance is variable and disease is usually present as a progressive sensory motor neuropathy with autonomic manifestations.^40^ Without treatment, the disease is usually progressive and fatal about 10 to 13 years after the onset of symptoms. Men and women are equally affected.^41^ On the other hand, prototypical hATTR-CM usually has a later onset than TTR-FAP, with symptoms occurring mostly after age 60.^42^ It manifests as unexplained left ventricular hypertrophy leading to restrictive cardiomyopathy, HF, atrial fibrillation, and conduction abnormalities.^43,44,45^

## Specific TTR Pathogenic Variants

### Val30Met

Discovered in the 1950s, Val30Met was the first TTR variant, initially identified in Portugal and later in Sweden and Japan. It typically causes FAP, resulting from a point mutation causing substitution of methionine for valine at position 30 of the mature protein.^46^ Family history is usually positive in endemic areas because inheritance is autosomal dominant.^10^ Val30Met can manifest as early- or late-onset disease and, as a result of this heterogeneity, may be difficult to diagnose.^10,46^ Although cardiac involvement is late among those with endemic disease, it tends to be more prominent in nonendemic areas and can lead to conduction abnormalities.^7^ Late-onset Val30Met manifests as ATTRv-CM as the disease progresses.^47^ The Val30Met variant is the most common mutation in several regions around the world; the largest group of individuals carrying this TTR variant may be found in northern Sweden with a prevalence of 4%,^48,49^ with a gene carrier frequency of 1:538 in northern Portugal.^50^ The usual age of disease onset in Portugal, Brazil, and Japan is in the third and fourth decade of life. In Portugal and Japan, more than 90% of TTR Val30Met gene carriers are symptomatic. In endemic areas of Portugal, the penetrance is high, with approximately 80% developing disease by age 50 years.^51,52^ Despite the high frequency of the Val30Met mutation in the endemic areas of Sweden (4%), there is relatively low penetrance: 11% by age 50, with older age of onset and a slower disease progression than in Portugal.^37^ Female gender seems to provide some protection from myocardial involvement, at least before the onset of menopause.^53^

### Val122Ile

Val122Ile is the most common *TTR* mutation in the US, resulting from a valine-to-isoleucine substitution at position 122 of the mature protein.^27,28^ It has a prevalence of approximately 3.4% among African Americans, with a low penetrance that is as yet undefined. It presents with a cardiac phenotype, usually earlier than ATTRwt, typically during or after the sixth decade of life, and this variant is associated with more neurologic symptoms than ATTRwt. The Val122Ile mutation has been found in 10% of African Americans over the age of 65 years who have severe HF.^28^ The mutation ultimately leads to misfolding of free TTR monomers, with an age-dependent phenotypic penetrance. After age 70, carriers show a higher frequency of HF and increased mortality.^22,28^

### Thr60Ala

Thr60Ala is the most common variant in the British and Irish populations, presenting with a mixed phenotype of HF and polyneuropathy, including severe autonomic neuropathy.^31,32^ Neurologic manifestations are more prevalent at an earlier stage, while cardiac involvement is usually present on diagnosis and is a major determinant of its poor prognosis. In the US, carriers of this pathogenic variant are more commonly in the Appalachian region with Irish ancestry, likely due to a common founder effect.

### Thr119Met

Thr119Met is a variant known to stabilize the transthyretin tetramer and has been associated with more favorable outcomes.^5^ This rare coding sequence is kinetically very stable and has been reported to be associated with increased longevity in a large Danish population study.^54,55^ When it occurs in trans with the Val30Met mutation, it counteracts the destabilizing effect and delays or prevents ATTR.^55^

## Prognosis of ATTR-CM

With advancements in diagnostic imaging and the emergence of novel TTR-targeted therapies, the prognosis of ATTR-CM is improving. However, for those who remain undiagnosed or cannot access novel treatments, there remains significant morbidity and mortality.

### Staging Systems

To help prognosticate at the time of diagnosis, two staging systems are widely used for patients with ATTR-CM, one from Mayo Clinic and one from the UK National Amyloidosis Centre.^14,56^ It should be pointed out, however, that these were developed prior to the more widespread use of disease-modifying therapies, which are associated with notable improvements in survival.

For the Mayo risk model,^14^ a cohort of ATTRwt patients were classified into three prognostic stages based on elevations of troponin T (> 0.05 ng/mL) and N-terminal proB-type natriuretic peptide (NT-proBNP) (> 3,000 pg/mL) biomarkers. Stage I included patients with no elevated biomarkers, Stage II with elevation in one biomarker, and Stage III with elevations in both biomarkers. Patients classified in Stage III had significantly worse median survival compared with those classified in Stage I. Median survival for patients classified in Stages I, II, and III was 66, 40, and 20 months, respectively.

For the UK risk model,^56^ the study cohort included both wild-type and hereditary ATTR variants, including the more common Val122Ile variant. Again, three prognostic stages were defined based on renal dysfunction (eGFR threshold of < 45 mL/min/1.73 m^2^) and elevations in NT-proBNP (> 3,000 pg/mL). Stage I included patients with no elevation in NT-proBNP and eGFR above or equal to the threshold, Stage III with elevation in NT-proBNP and eGFR below the threshold, and Stage II included the remainder of the cohort (either elevated NT-proBNP or low eGFR alone). Outcomes were significantly worse with higher stages. Median survival for patients classified in Stage I, II, and III was 69.2, 46.7, and 24.1 months, respectively. This finding remained significant even after controlling for different genotypes and was further validated in a separate cohort of ATTR-CM patients.

More recently, a group from Columbia University evaluated the additive prognostic value of diuretic dose and New York Heart Association (NYHA) functional class to these preexisting risk staging systems.^57^ Daily diuretic dosing was categorized into furosemide equivalents and assigned to a point system, with 0 points for 0 mg/kg, 1 point for > 0 to 0.5 mg/kg, 2 points for > 0.5 to 1 mg/kg, and 3 points for > 1 mg/kg. A similar point system was used for NYHA functional class with 1 point per class ranging from 1 to 4 points. The addition of these variables significantly improved the accuracy of the preexisting risk staging systems, increasing the area under the curve for the Mayo risk model from 0.693 to 0.798 and the UK risk model from 0.711 to 0.813 for all-cause mortality.

It should be noted that while these biomarkers tend to be readily available and should be considered during the initial evaluation for all patients with ATTR-CM, analogous blood tests—including high-sensitivity troponin assays and B-type natriuretic peptide (BNP)—circulate at different concentrations in plasma. A separate biologically active hormone from NT-proBNP, BNP has a shorter half-life and can be affected by use of the angiotensin receptor-neprilysin inhibitor (ARNI) class of HF therapeutics.^58^ As such, high-sensitivity troponin and BNP concentrations are not interchangeable biomarkers for the previously described staging systems.

### High Risk Clinical Features

Clinical features in patients with ATTR-CM, including functional status, certain comorbid conditions, and specific genotypes, have been independently associated with increased mortality. Worsening survival among those with ATTRwt-CM has been observed with declining NYHA functional classes.^59^ Additionally, NYHA functional class III or IV was identified as an independent risk factor for adverse cardiovascular outcomes that include the development of conduction disease, HF hospitalization, and stroke.^60,61^ Results from the Transthyretin Amyloidosis Cardiomyopathy Clinical Trial (ATTR-ACT) indicated diminished benefit from the TTR stabilizing agent, tafamidis, in patients with NYHA functional class III compared to those with NYHA class I or II disease, highlighting the need for early detection of ATTR-CM.^62^

Atrial fibrillation is more common in ATTRwt compared with ATTRv and is present in up to 70% of ATTRwt patients.^63^ The presence of atrial fibrillation, atrial flutter, or atrioventricular block is associated with worse prognosis. Although not independently associated with worse mortality compared to those with sinus rhythm, ATTR-CM patients with atrial fibrillation were more likely to have severe HF symptoms (NYHA class III or IV) and a higher frequency of renal dysfunction.^64,65^ Furthermore, ATTR-CM patients are at increased risk for thromboembolism,^66^ with one study examining cardioversion outcomes for atrial arrhythmias, noting a significantly high procedural cancellation rate mainly due to the presence of intracardiac thrombus (up to 30%) despite adequate anticoagulation.^67^ Another study of patients with ATTR-CM and atrial fibrillation noted no association between CHA2DS2-VASc score and the presence of left atrial appendage thrombus.^68^

Conduction system disease often develops in patients with ATTR-CM, and requirement of a pacemaker has been associated with worse survival.^59^ In a recent study examining ATTR-CM patients with implantable cardiac devices, those with a higher RV pacing burden (> 40%) experienced worsening HF, left ventricular ejection fraction (LVEF), and mitral regurgitation severity.^69^ In contrast, those with biventricular pacing noted improvements in LVEF, NYHA class, and severity of mitral regurgitation. These findings suggest that biventricular pacing should be used in patients with ATTR-CM and an indication for pacing.^70^ Ventricular arrhythmias are common in ATTR-CM, most commonly nonsustained ventricular tachycardia (up to 74%). However, placement of an implantable cardioverter defibrillator (ICD) for primary or secondary prevention has not been shown to improve survival.^71^ Currently, ICD implantation is of unclear benefit in this population and will need prospective studies to determine their utility as medical therapies for ATTR-CM that may improve survival and alter disease course.

Finally, specific genotypes have been identified as carrying a worse prognosis compared with other familial variants and ATTRwt. In a large ATTR-CM cohort, patients with the Val122Ile variant had significantly worse median survival compared to those with non-Val122Ile variants or wild-type disease.^72^

### Morbidity

While not associated with survival, neuropathic involvement and functional decline in patients with ATTR-CM can add significant morbidity. This is best illustrated by studies of TTR-targeted therapies and their respective improvements in neuropathic impairment scores, functional assessments, and quality of life questionnaires. The early studies on the clinical applications of tafamidis indicated its ability to stabilize the TTR tetramer and delay peripheral neurologic impairment.^8,73^ Furthermore, results from the ATTR-ACT trial noted significant improvement in several key secondary end points, including distance walked during the 6-minute walk test and KCCQ-OS score, a measure of quality of life, with differences first observed at 6 months.^62^ Results from the APOLLO study—a phase 3 trial for patients with familial amyloid polyneuropathy randomized to placebo versus patisiran, a small-interfering RNA-based drug targeting hepatic synthesis of TTR—noted significant improvements in neuropathy impairment scores, 10-meter walk test, and quality of life.^74^ Treatment with another TTR “silencer,” inotersen, led to similar outcomes, although inotersen use was complicated by serious adverse events with glomerulonephritis (3%) and severe thrombocytopenia (3%) in this trial.^75^ Both patisiran and inotersen are approved by the US Food and Drug Administration for TTR-FAP but not indicated for ATTRwt or ATTRv-CM without neuropathy. The CARDIO-TTRansform trial is currently randomizing participants with ATTR-CM to receive placebo versus eplontersen, a ligand-conjugated investigational antisense medicine designed to reduce the production of transthyretin. The HELIOS-B study is a phase 3 trial of vutrisiran, an investigational therapeutic for patients with ATTR-CM similarly designed to reduce production of transthyretin with a longer acting effect.

In summary, long-term prognostic data are limited in the era of TTR-targeted therapeutics for patients with ATTR-CM. Current staging systems were published prior to the development and more widespread use of these novel agents. As such, studies indicate that in patients with ATTR-CM, those receiving treatment have significantly improved morbidity and mortality, especially if diagnosed at an early stage of disease.

## Methods

Two online databases were explored, Google Scholar and PubMed, to retrieve relevant articles and published studies, and different keywords and search terms were used to find those most relevant. These keywords were “amyloidosis,” “transthyretin amyloidosis,” “epidemiology of ATTR amyloidosis,” “genetics of ATTR amyloidosis,” “prognosis of ATTR amyloidosis,” “TTR variants,” and “ATTR amyloidosis AND treatments.”

Exclusion and inclusion criteria were applied to the complete contents of searched articles to identify the most relevant studies. Only randomized controlled trials, meta-analyses, observational studies with controls, case series, review articles, and reports were included. Cross references of retrieved articles were also explored to find the relevant articles. Articles from non-peer review journals, editorials, conference papers, abstracts, and letters were excluded.

## Conclusion

Our recognition of the epidemiology of ATTR-CM, a disease that was once considered rare, is significantly changing due to increased awareness among healthcare providers and broader use of cardiac scintigraphy. There is considerable heterogeneity in the clinical presentation, and phenotypic heterogeneity is dependent on both genetic and environmental factors. Early diagnoses, recognition of the early stages of cardiac infiltration, and initiation of treatment are essential to optimize long-term prognosis.

## Key Points

- Phenotypic heterogeneity is linked to specific pathogenic variants in transthyretin, geography, and type of disease (endemic versus nonendemic).
- Early diagnosis is essential for effective treatment and favorable prognosis.
- Patients receiving treatment for transthyretin amyloid cardiomyopathy have significantly improved morbidity and mortality compared with historical data on patients without disease-modifying therapies.

## References

[1] Wechalekar AD, Gillmore JD, Hawkins PN. Systemic amyloidosis. Lancet. 2016 Jun 25;387(10038):2641-2654. doi: 10.1016/S0140-6736(15)01274-X 26719234

[2] Kristen AV, Ajroud-Driss S, Conceição I, et al. Patisiran, an RNAi therapeutic for the treatment of hereditary transthyretin-mediated amyloidosis. Neurodegener Dis Manag. 2019 Feb;9(1):5-23. doi: 10.2217/nmt-2018-0033 30480471

[3] Finsterer J, Iglseder S, Wanschitz J, et al. Hereditary transthyretin-related amyloidosis. Acta Neurol Scand. 2019 Feb;139(2):92-105. doi: 10.1111/ane.13035 30295933

[4] Manolis AS, Manolis AA, Manolis TA, Melita H. Cardiac amyloidosis: An underdiagnosed/underappreciated disease. Eur J Intern Med. 2019 Sep;67:1-13. doi: 10.1016/j.ejim.2019.07.022 31375251

[5] Yamamoto H, Yokochi T. Transthyretin cardiac amyloidosis: an update on diagnosis and treatment. ESC Heart Fail. 2019 Dec;6(6):1128-1139. doi: 10.1002/ehf2.12518 31553132 PMC6989279

[6] Coelho T, Maurer MS, Suhr OB. THAOS – The Transthyretin Amyloidosis Outcomes Survey: initial report on clinical manifestations in patients with hereditary and wild-type transthyretin amyloidosis. Curr Med Res Opin. 2013 Jan;29(1):63-76. doi: 10.1185/03007995.2012.754348 23193944

[7] Stakos DA, Stamatelopoulos K, Bampatsias D, et al. The Alzheimer’s Disease Amyloid-Beta Hypothesis in Cardiovascular Aging and Disease: JACC Focus Seminar. J Am Coll Cardiol. 2020 Mar 3;75(8):952-967. doi: 10.1016/j.jacc.2019.12.033 32130931 PMC7042886

[8] Coelho T, Merlini G, Bulawa CE, et al. Mechanism of Action and Clinical Application of Tafamidis in Hereditary Transthyretin Amyloidosis. Neurol Ther. 2016 Jun;5(1):1-25. doi: 10.1007/s40120-016-0040-x 26894299 PMC4919130

[9] Maurer MS, Elliott P, Comenzo R, Semigran M, Rapezzi C. Addressing Common Questions Encountered in the Diagnosis and Management of Cardiac Amyloidosis. Circulation. 2017 Apr 4;135(14):1357-1377. doi: 10.1161/CIRCULATIONAHA.116.024438 28373528 PMC5392416

[10] Rapezzi C, Lorenzini M, Longhi S, et al. Cardiac amyloidosis: the great pretender. Heart Fail Rev. 2015 Mar;20(2):117-24. doi: 10.1007/s10741-015-9480-0 25758359

[11] Palladini G, Merlini G. Systemic amyloidoses: what an internist should know. Eur J Intern Med. 2013 Dec;24(8):729-39. doi: 10.1016/j.ejim.2013.10.007 24262289

[12] Lauppe RE, Hansen JL, Gerdesköld C, et al. Nationwide prevalence and characteristics of transthyretin amyloid cardiomyopathy in Sweden. Open Heart. 2021 Oct;8(2):e001755. doi: 10.1136/openhrt-2021-001755 34645699 PMC8515473

[13] Porcari A, Merlo M, Rapezzi C, Sinagra G. Transthyretin amyloid cardiomyopathy: An uncharted territory awaiting discovery. Eur J Intern Med. 2020 Dec;82:7-15. doi: 10.1016/j.ejim.2020.09.025 PMC7534738 33032855

[14] Grogan M, Scott CG, Kyle RA, et al. Natural History of Wild-Type Transthyretin Cardiac Amyloidosis and Risk Stratification Using a Novel Staging System. J Am Coll Cardiol. 2016 Sep 6;68(10):1014-20. doi: 10.1016/j.jacc.2016.06.033 27585505

[15] Rapezzi C, Quarta CC, Riva L, et al. Transthyretin-related amyloidoses and the heart: a clinical overview. Nat Rev Cardiol. 2010 Jul;7(7):398-408. doi: 10.1038/nrcardio.2010.67 20479782

[16] Castaño A, Narotsky DL, Hamid N, et al. Unveiling transthyretin cardiac amyloidosis and its predictors among elderly patients with severe aortic stenosis undergoing transcatheter aortic valve replacement. Eur Heart J. 2017 Oct 7;38(38):2879-2887. doi: 10.1093/eurheartj/ehx350 29019612 PMC5837725

[17] Papoutsidakis N, Miller EJ, Rodonski A, Jacoby D. Time Course of Common Clinical Manifestations in Patients with Transthyretin Cardiac Amyloidosis: Delay From Symptom Onset to Diagnosis. J Card Fail. 2018 Feb;24(2):131-133. doi: 10.1016/j.cardfail.2017.12.005 29305186

[18] Tanskanen M, Peuralinna T, Polvikoski T, et al. Senile systemic amyloidosis affects 25% of the very aged and associates with genetic variation in alpha2-macroglobulin and tau: a population-based autopsy study. Ann Med. 2008;40(3):232-9. doi: 10.1080/07853890701842988 18382889

[19] Virani SS, Alonso A, Benjamin EJ, et al. Heart disease and stroke statistics—2020 update: a report from the American Heart Association. Circulation. 2020 Mar 3;141(9):e139-596. doi: 10.1161/CIR.0000000000000757 31992061

[20] Mohammed SF, Mirzoyev SA, Edwards WD, et al. Left ventricular amyloid deposition in patients with heart failure and preserved ejection fraction. JACC Heart Fail. 2014 Apr;2(2):113-22. doi: 10.1016/j.jchf.2013.11.004 24720917 PMC3984539

[21] Roger VL. Epidemiology of Heart Failure: A Contemporary Perspective. Circ Res. 2021 May 14;128(10):1421-1434. doi: 10.1161/CIRCRESAHA.121.318172 33983838

[22] Ruberg FL, Berk JL. Transthyretin (TTR) cardiac amyloidosis. Circulation. 2012 Sep 4;126(10):1286-300. doi: 10.1161/CIRCULATIONAHA.111.078915 22949539 PMC3501197

[23] Dwulet FE, Benson MD. Primary structure of an amyloid prealbumin and its plasma precursor in a heredofamilial polyneuropathy of Swedish origin. Proc Natl Acad Sci U S A. 1984 Feb;81(3):694-8. doi: 10.1073/pnas.81.3.694 6583672 PMC344901

[24] Tsuzuki T, Mita S, Araki S, Shimada K. Structure of the human prealbumin gene. J Biol Chem. 1985 Oct 5;260(22):12224-7. doi: 10.1016/S0021-9258(17)39013-0 2995367

[25] Conceição I, Damy T, Romero M, et al. Early diagnosis of ATTR amyloidosis through targeted follow-up of identified carriers of TTR gene mutations. Amyloid. 2019 Mar;26(1):3-9. doi: 10.1080/13506129.2018.1556156 30793974

[26] Brown EE, Fajardo J, Judge DP. Positive family history decreases diagnosis time by over 200. Amyloid. 2019;26(sup1):17. doi: 10.1080/13506129.2019.1582484 31343334

[27] Maurer MS, Hanna M, Grogan M, et al. Genotype and Phenotype of Transthyretin Cardiac Amyloidosis: THAOS (Transthyretin Amyloid Outcome Survey). J Am Coll Cardiol. 2016 Jul 12;68(2):161-72. doi: 10.1016/j.jacc.2016.03.596 27386769 PMC4940135

[28] Jacobson DR, Alexander AA, Tagoe C, Buxbaum JN. Prevalence of the amyloidogenic transthyretin (TTR) V122I allele in 14 333 African-Americans. Amyloid. 2015;22(3):171-4. doi: 10.3109/13506129.2015.1051219 26123279

[29] Kirov A, Sarafov S, Pavlova Z, et al. Founder effect of the Glu89Gln TTR mutation in the Bulgarian population. Amyloid. 2019 Dec;26(4):181-185. doi: 10.1080/13506129.2019.1634539 31353960

[30] Damy T, Kristen AV, Suhr OB, et al. Transthyretin cardiac amyloidosis in continental Western Europe: an insight through the Transthyretin Amyloidosis Outcomes Survey (THAOS). Eur Heart J. 2019 Apr 1;ehz173. doi: 10.1093/eurheartj/ehz173 30938420 PMC8825236

[31] Sattianayagam PT, Hahn AF, Whelan CJ, et al. Cardiac phenotype and clinical outcome of familial amyloid polyneuropathy associated with transthyretin alanine 60 variant. Eur Heart J. 2012 May;33(9):1120-7. doi: 10.1093/eurheartj/ehr383 21992998

[32] Reilly MM, Staunton H, Harding AE. Familial amyloid polyneuropathy (TTR ala 60) in north west Ireland: a clinical, genetic, and epidemiological study. J Neurol Neurosurg Psychiatry. 1995 Jul;59(1):45-9. doi: 10.1136/jnnp.59.1.45 7608709 PMC1073600

[33] Buxbaum JN, Ruberg FL. Transthyretin V122I (pV142I)* cardiac amyloidosis: an age-dependent autosomal dominant cardiomyopathy too common to be overlooked as a cause of significant heart disease in elderly African Americans. Genet Med. 2017 Jul;19(7):733-742. doi: 10.1038/gim.2016.200 28102864 PMC5509498

[34] Ando Y, Coelho T, Berk JL, et al. Guideline of transthyretin-related hereditary amyloidosis for clinicians. Orphanet J Rare Dis. 2013 Feb 20;8:31. doi: 10.1186/1750-1172-8-31 23425518 PMC3584981

[35] Uemichi T, Uitti RJ, Koeppen AH, Donat JR, Benson MD. Oculoleptomeningeal amyloidosis associated with a new transthyretin variant Ser64. Arch Neurol. 1999 Sep;56(9):1152-5. doi: 10.1001/archneur.56.9.1152 10488818

[36] Trachtenberg BH, Shah SK, Nussbaum RL, Bristow SL, Malladi R, Vatta M. Presence of the V122I Variant of Hereditary Transthyretin-Mediated Amyloidosis Among Self-Reported White Individuals in a Sponsored Genetic Testing Program. Circ Genom Precis Med. 2021 Oct;14(5):e003466. doi: 10.1161/CIRCGEN.121.003466 34461735

[37] Parman Y, Adams D, Obici L, et al.; European Network for TTR-FAP (ATTReuNET). Sixty years of transthyretin familial amyloid polyneuropathy (TTR-FAP) in Europe: where are we now? A European network approach to defining the epidemiology and management patterns for TTR-FAP. Curr Opin Neurol. 2016 Feb;29 Suppl 1(Suppl 1):S3-S13. doi: 10.1097/WCO.0000000000000288 26734951 PMC4739317

[38] Bonaïti B, Alarcon F, Bonaïti-Pellié C, Planté-Bordeneuve V. Parent-of-origin effect in transthyretin related amyloid polyneuropathy. Amyloid. 2009;16(3):149-50. doi: 10.1080/13506120903093944 19626480

[39] Cuddy SAM, Falk RH. Amyloidosis as a Systemic Disease in Context. Can J Cardiol. 2020 Mar;36(3):396-407. doi: 10.1016/j.cjca.2019.12.033 32145867

[40] Vio R, Angelini A, Basso C, et al. Hypertrophic Cardiomyopathy and Primary Restrictive Cardiomyopathy: Similarities, Differences and Phenocopies. 2021 May 1;10(9):1954. doi: 10.3390/jcm10091954 PMC8125617 34062949

[41] Gertz MA. Hereditary ATTR amyloidosis: burden of illness and diagnostic challenges. Am J Manag Care. 2017 Jun;23(7 Suppl):S107-S112 28978215

[42] Judge DP, Heitner SB, Falk RH, et al. Transthyretin Stabilization by AG10 in Symptomatic Transthyretin Amyloid Cardiomyopathy. J Am Coll Cardiol. 2019 Jul 23;74(3):285-295. doi: 10.1016/j.jacc.2019.03.012 30885685

[43] González-López E, Gallego-Delgado M, Guzzo-Merello G, et al. Wild-type transthyretin amyloidosis as a cause of heart failure with preserved ejection fraction. Eur Heart J. 2015 Oct 7;36(38):2585-94. doi: 10.1093/eurheartj/ehv338 26224076

[44] Oerlemans MIFJ, Rutten KHG, Minnema MC, Raymakers RAP, Asselbergs FW, de Jonge N. Cardiac amyloidosis: the need for early diagnosis. Neth Heart J. 2019 Nov;27(11):525-536. doi: 10.1007/s12471-019-1299-1 31359320 PMC6823341

[45] Alexander KM, Maurer MS, Fergus IV. Cardiac Amyloid Heart Disease in Racial/Ethnic Minorities: Focus on Transthyretin Amyloid Cardiomyopathy. In: Ferdinand KC, Taylor HA Jr. Rodriguez CJ, editors. Cardiovascular Disease in Racial and Ethnic Minority Populations. New York, NY: Springer International Publishing; 2021. p. 201-215. doi: 10.1007/978-3-030-81034-4_17

[46] Schwartzlow C, Kazamel M. Hereditary Transthyretin Amyloidosis: Clinical Presentation and Management Updates. J Clin Neuromuscul Dis. 2020 Mar;21(3):144-156. doi: 10.1097/CND.0000000000000270 32073460

[47] Manganelli F, Fabrizi GM, Luigetti M, Mandich P, Mazzeo A, Pareyson D. Hereditary transthyretin amyloidosis overview. Neurol Sci. 2020 Nov 14. doi: 10.1007/s10072-020-04889-2 PMC9780126 33188616

[48] Holmgren G, Costa PM, Andersson C, et al. Geographical distribution of TTR met30 carriers in northern Sweden: discrepancy between carrier frequency and prevalence rate. J Med Genet. 1994 May;31(5):351-4. doi: 10.1136/jmg.31.5.351 8064809 PMC1049863

[49] Hellman U, Alarcon F, Lundgren H-E, Suhr OB, Bonaiti-Pellié C, Planté-Bordeneuve V. Heterogeneity of penetrance in familial amyloid polyneuropathy, ATTR Val30Met, in the Swedish population. Amyloid. 2008 Sep;15(3):181-6. doi: 10.1080/13506120802193720 18925456 PMC2738945

[50] Conceição I. Clinical features of TTR-FAP in Portugal. Amyloid. 2012 Jun;19 Suppl 1:71-2. doi: 10.3109/13506129.2012.673184 22480206

[51] Jacobson DR, Pastore RD, Yaghoubian R, et al. Variant-sequence transthyretin (isoleucine 122) in late-onset cardiac amyloidosis in black Americans. N Engl J Med. 1997 Feb 13;336(7):466-73. doi: 10.1056/NEJM199702133360703 9017939

[52] Soares ML, Coelho T, Sousa A, et al. Haplotypes and DNA sequence variation within and surrounding the transthyretin gene: genotype-phenotype correlations in familial amyloid polyneuropathy (V30M) in Portugal and Sweden. Eur J Hum Genet. 2004 Mar;12(3):225-37. doi: 10.1038/sj.ejhg.5201095 14673473

[53] Rapezzi C, Riva L, Quarta CC, et al. Gender-related risk of myocardial involvement in systemic amyloidosis. Amyloid. 2008 Mar;15(1):40-8. doi: 10.1080/13506120701815373 18266120

[54] Liang D. The potency of AG10 in stabilizing transthyretin is driven by interactions mimicking the disease-suppressing T119M variant [dissertation]. Stockton, California: University of the Pacific; 2019. 57 p.

[55] Hammarström P, Schneider F, Kelly JW. Trans-suppression of misfolding in an amyloid disease. Science. 2001 Sep 28;293(5539):2459-62. doi: 10.1126/science.1062245 11577236

[56] Gillmore JD, Damy T, Fontana M, et al. A new staging system for cardiac transthyretin amyloidosis. Eur Heart J. 2018 Aug 7;39(30):2799-2806. doi: 10.1093/eurheartj/ehx589 29048471

[57] Cheng RK, Levy WC, Vasbinder A, et al. Diuretic Dose and NYHA Functional Class Are Independent Predictors of Mortality in Patients With Transthyretin Cardiac Amyloidosis. JACC CardioOncol. 2020 Sep;2(3):414-424. doi: 10.1016/j.jaccao.2020.06.007 33073249 PMC7561022

[58] Myhre PL, Vaduganathan M, Claggett B, et al. B-Type Natriuretic Peptide During Treatment With Sacubitril/Valsartan: The PARADIGM-HF Trial. J Am Coll Cardiol. 2019 Mar 26;73(11):1264-1272. doi: 10.1016/j.jacc.2019.01.018 30846338 PMC7955687

[59] Pinney JH, Whelan CJ, Petrie A, et al. Senile systemic amyloidosis: clinical features at presentation and outcome. J Am Heart Assoc. 2013 Apr 22;2(2):e000098. doi: 10.1161/JAHA.113.000098 23608605 PMC3647259

[60] Rapezzi C, Merlini G, Quarta CC, et al. Systemic cardiac amyloidoses: disease profiles and clinical courses of the 3 main types. Circulation. 2009 Sep 29;120(13):1203-12. doi: 10.1161/CIRCULATIONAHA.108.843334 19752327

[61] Galat A, Rosso J, Guellich A, et al. Usefulness of (99m)Tc-HMDP scintigraphy for the etiologic diagnosis and prognosis of cardiac amyloidosis. Amyloid. 2015;22(4):210-20. doi: 10.3109/13506129.2015.1072089 26465835

[62] Maurer MS, Schwartz JH, Gundapaneni B, et al. Tafamidis Treatment for Patients with Transthyretin Amyloid Cardiomyopathy. N Engl J Med. 2018 Sep 13;379(11):1007-1016. doi: 10.1056/NEJMoa1805689 30145929

[63] Sanchis K, Cariou E, Colombat M, et al. Atrial fibrillation and subtype of atrial fibrillation in cardiac amyloidosis: clinical and echocardiographic features, impact on mortality. Amyloid. 2019 Sep;26(3):128-138. doi: 10.1080/13506129.2019.1620724 31172799

[64] Longhi S, Quarta CC, Milandri A, et al. Atrial fibrillation in amyloidotic cardiomyopathy: prevalence, incidence, risk factors and prognostic role. Amyloid. 2015;22(3):147-55. doi: 10.3109/13506129.2015.1028616 25997105

[65] Mints YY, Doros G, Berk JL, Connors LH, Ruberg FL. Features of atrial fibrillation in wild-type transthyretin cardiac amyloidosis: a systematic review and clinical experience. ESC Heart Fail. 2018 Oct;5(5):772-779. doi: 10.1002/ehf2.12308 29916559 PMC6165925

[66] Feng DaLi, Edwards WD, Oh JK, et al. Intracardiac thrombosis and embolism in patients with cardiac amyloidosis. Circulation. 2007 Nov 20;116(21):2420-6. doi: 10.1161/CIRCULATIONAHA.107.697763 17984380

[67] El-Am EA, Dispenzieri A, Melduni RM, et al. Direct Current Cardioversion of Atrial Arrhythmias in Adults With Cardiac Amyloidosis. J Am Coll Cardiol. 2019 Feb 12;73(5):589-597. doi: 10.1016/j.jacc.2018.10.079 30732713 PMC6378685

[68] Donnellan E, Elshazly MB, Vakamudi S, et al. No Association Between CHADS-VASc Score and Left Atrial Appendage Thrombus in Patients With Transthyretin Amyloidosis. JACC Clin Electrophysiol. 2019 Dec;5(12):1473-1474. doi: 10.1016/j.jacep.2019.10.013 31857048

[69] Donnellan E, Wazni OM, Saliba WI, et al. Cardiac devices in patients with transthyretin amyloidosis: Impact on functional class, left ventricular function, mitral regurgitation, and mortality. J Cardiovasc Electrophysiol. 2019 Nov;30(11):2427-2432. doi: 10.1111/jce.14180 31515942

[70] Donnellan E, Wazni OM, Hanna M, Kanj M, Saliba WI, Jaber WA. Cardiac Resynchronization Therapy for Transthyretin Cardiac Amyloidosis. J Am Heart Assoc. 2020 Jul 21;9(14):e017335. doi: 10.1161/JAHA.120.017335 32633203 PMC7660724

[71] Giancaterino S, Urey MA, Darden D, Hsu JC. Management of Arrhythmias in Cardiac Amyloidosis. JACC Clin Electrophysiol. 2020 Apr;6(4):351-361. doi: 10.1016/j.jacep.2020.01.004 32327068

[72] Lane T, Fontana M, Martinez-Naharro A, et al. Natural History, Quality of Life, and Outcome in Cardiac Transthyretin Amyloidosis. Circulation. 2019 Jul 2;140(1):16-26. doi: 10.1161/CIRCULATIONAHA.118.038169 31109193

[73] Coelho T, Maia LF, da Silva AM, et al. Tafamidis for transthyretin familial amyloid polyneuropathy: a randomized, controlled trial. Neurology. 2012 Aug 21;79(8):785-92. doi: 10.1212/WNL.0b013e3182661eb1 22843282 PMC4098875

[74] Adams D, Gonzalez-Duarte A, O’Riordan WD, et al. Patisiran, an RNAi Therapeutic, for Hereditary Transthyretin Amyloidosis. N Engl J Med. 2018 Jul 5;379(1):11-21. doi: 10.1056/NEJMoa1716153 29972753

[75] Benson MD, Waddington-Cruz M, Berk JL, et al. Inotersen Treatment for Patients with Hereditary Transthyretin Amyloidosis. N Engl J Med. 2018 Jul 5;379(1):22-31. doi: 10.1056/NEJMoa1716793 29972757 PMC12611561

